# A Retrospective Study on Using a Novel Single Needle Cone Puncture Approach for the Iodine-125 Seed Brachytherapy in Treating Patients With Thoracic Malignancy

**DOI:** 10.3389/fonc.2021.640131

**Published:** 2021-05-31

**Authors:** Fenge Li, Liping Wang, Yixiang Zhang, Weihong Feng, Tao Ju, Zaiping Liu, Zhenglu Wang, Xueming Du

**Affiliations:** ^1^ Department of Oncology, Tianjin Beichen Hospital, Tianjin, China; ^2^ Department of Melanoma Oncology, University of Texas M.D. Anderson Cancer Center, Houston, TX, United States; ^3^ Department of Oncology, Weifang People’s Hospital, Weifang, China; ^4^ Pulmonary Medicine, Weifang People’s Hospital, Weifang, China; ^5^ Department of Pathology and Laboratory Medicine, IWK Women’s and Children’s Health Center, Halifax, NS, Canada; ^6^ Pathology Department, Tianjin First Central Hospital, Tianjin, China

**Keywords:** radioactive iodine-125 brachytherapy, single needle cone puncture, thoracic malignancy, life-threatening symptom, survival

## Abstract

**Background:**

Patients with progressive thoracic malignancy characterized by large irregular tumors with necrosis and life-threatening symptoms lack effective treatments. We set out to develop a single needle cone puncture method for the Iodine-125 seed (SNCP-^125^I) brachytherapy, and aim to report the initial results.

**Methods:**

294 patients with advanced thoracic malignancy were treated with local SNCP-^125^I brachytherapy between March 2009 and July 2020, followed by thorough evaluation of clinical outcome, overall survival (OS), progression-free survival (PFS) and procedure-related complications after treatment.

**Results:**

The overall response rate (ORR) among the treated patients was 81.0% (238/294). Life-threatening symptoms due to tumor oppression, hemoptysis and large irregular tumor with necrosis were successfully alleviated after the SNCP-^125^I treatment with a remission rate at 91% to 94%. The median OS and PFS were 13.6 months and 5.8 months, respectively. Procedure-related side effects including pneumothorax (32/294), blood-stained sputum (8/294), subcutaneous emphysema (10/294), puncture site bleeding (16/294) and chest pain (6/294) were observed. Patients who were able to follow with chemotherapy or immunotherapy experienced extended OS and PFS, as compared with patients who opted to receive hospice care (16.5 months Vs. 11.2 months). Further pathological and immunological analysis showed that SNCP-^125^I induced tumor lymphocytes infiltration and long-term tumor necrosis.

**Conclusion:**

SNCP-^125^I brachytherapy effectively eliminates life-threatening symptoms due to local tumor oppression, hemoptysis and large irregular and necrotic tumors in patients with unresectable chest malignancy and significantly induces local tumor regression. SNCP-^125^I brachytherapy combines with chemotherapy significantly prolong OS and PFS compare with SNCP-^125^I brachytherapy alone.

## Introduction

Successful treatment of local incurable thoracic malignancy, including lung squamous cell carcinoma, metastatic esophageal cancer, and unresectable malignant thymoma, has been hampered by the lack of clinically effective regimens. This represents a particular obstacle for patients who experience rapid tumor progression as a result of large irregular and necrotic tumors, hemoptysis and lethal symptoms, but are not susceptible to local interventiondue to limited access to the site of tumors. As such, despite systemic therapeutic treatment, coupled with advanced imaging technology, local lesions still reoccur and develop. Further, tumor pathology is closely associated with disease progression and therapy responses (1–3). Patients with squamous cell carcinoma exhibit unfavorable outcomes when treated with chemotherapy, immunotherapy, and molecularly targeted therapy, especially for central lung cancer and mediastinal tumors that are frequently surrounded by large blood vessels and trachea. This elevates the potential risk for performing local puncture operations. It should be further noted that the tumors often progress aggressively, thereby constricting adjacent trachea, blood vessels, heart and esophagus and leading to lethal dyspnea, hemoptysis, dysphagia and superior vena cava syndrome, which primarily accounts for the mortality in cancer patients (4–7). Even though there is a low risk resulting from puncture operations for large tumors, the irregular necrosis, ribs, blood vessels and trachea often compromise the obstacle of the conventional interventional treatment. However, there is no effective approach to overcome these clinical challenges for patients with advanced malignancy, and the outcome after conventional treatment remains poor for those with squamous cell carcinomas of lung cancer, invasive thymic carcinoma, and chest metastatic tumors (8–10). Therefore, instead of hospice care, effective clinical strategies for these patients is urgently needed.

Computerized tomography (CT)-guided local radioactive Idione-125 (^125^I) seed brachytherapy has been widely used for various types of advanced cancers, including lung cancer, uveal melanoma, breast cancer, malignant gliomas and retroperitoneal malignant tumors (11–17). Treatment planning systems (TPS) are employed in CT-guided local radioactive ^125^I seed brachytherapy to ensure that the tumor site receives the maximum therapeutic dose while sparing surrounding tumor tissue, which represents one of the most effective approaches for maximum clinical benefit to the patient (18, 19). Conventional multiple-needle ^125^I brachytherapy method is only suitable for patients who can make required postures and have multiple parallel sites accessible for the puncture operation. However, this is not practical for patients with lung hilar and mediastinal tumors, because multiple needle puncture poses a great risk of damaging large blood vessels, and patients can quickly develop respiratory failure. In addition, additional criteria must be satisfied before the treatment. Foremost, the tumor lesion is located adjacent to the main bronchus, blood vessels, esophagus and heart. Secondly, the patient experiences the typical symptoms including dyspnea, hemoptysis, dysphagia, arrhythmia and superior vena cava syndrome as a result of local tumor oppression. Thirdly, the tumor is larger than 7 cm in size with irregular necrosis (20). Additional technical difficulties also prevents utilization and effectiveness of the multiple-needle brachytherapy, including lack of multiple puncture sites, uneven distribution of radioactive doses for large tumors, inability to maintain a posture for the operation, and extra poor performance status of these patients. Unfortunately, these patients are ultimately only able to choose hospice care with unfavorable survival outcomes.

To tackle this challenge, herein we report a single needle cone puncture method for the ^125^I seed brachytherapy, with which we implement a radioactive dose covering over 90% of the tumor volume using a single needle through one puncture site on the skin. We show greatly reduced risk associated with the operation and satisfied recovery rate upon treatment, especially in patients with lung hilar and mediastinum tumors. Utilizing this novel intervention method, we successfully and effectively treated thoracic malignancy patients with large irregular tumors and severe symptoms due to local tumor oppression and hemoptysis (ORR=81.0%). Detailed pathological and immunological analysis uncovers that ^125^I seed brachytherapy causes tumor necrosis within 15 to 20 days, associated with increased infiltration of tumor lymphocytes. Taken together, these results provide informative and critical insights into a new treatment strategy which helps to prolong survival for patients with advanced thoracic malignancy.

## Materials and Methods

### Patients Information and Characteristics

Two hundred and ninety-four patients with unresectable thoracic cancer showing severe symptoms resulting from tumor oppression received local radioactive ^125^I seed brachytherapy from March 2009 until July 2020 at Tianjin Beichen Hospital (Tianjin, China) were investigated in this study. This study conformed to the ethical guidelines of the 1975 Declaration of Helsinki and was approved by the Tianjin Anti-Cancer Association and ethics committee of Tianjin Beichen Hospital. All cases were selected according to the following inclusion criteria: 1) diagnosis with stage III/IV thoracic cancer with unresectable tumor; 2) confirmed malignancy with biopsy; 3) recurrence after conventional treatment including surgery, chemotherapy, immunotherapy and systematic radiotherapy, with no active treatment options available; 4) at least one type of the symptoms including dyspnea, hemoptysis, dysphagia, and super vena cava syndrome due to local tumor oppression, or tumor larger than 7 cm causing cachexia; 5) no history of chronic lung disease including pneumonia and Chronic Obstructive Pulmonary Disease (COPD); 6) no liver and kidney dysfunction, severe heart disease, impaired hematopoietic function or systemic infection. Patients were excluded if they did not meet the criteria above. Patient clinical characteristics are shown in **Table 1**. Of the 294 patients, there were 290 with squamous cell carcinoma and 4 with malignant thymic carcinoma (**Table 1** and **Supplemental Figure 1**). Of the 294 patients received ^125^I seeds, 117 patients received chemotherapy, two patients received immunotherapy 3 to 6 months after treatment, and 175 patients did not receive any other therapeutic treatments during follow-up. All patients’ data were collected from the hospital medical records which were described precisely, and those with incomplete information were excluded. Informed consents of all patients were obtained for the study.

**Table 1:** Clinical and demographic characteristics of patients at baseline.

Characteristics	Patients (n=294)
Gender (Male-Female)	223-71
Age (year, mean ± SD)	65.8 ± 9.6
Weight (kg, mean ± SD)	65.6 ± 9.1
Height (cm, mean ± SD)	169.2 ± 7.5
Smoking history, n (%)	225 (76.5)
Pleural effusion, n (%)	36(12.2)
Tumor size (cm, mean ± SD)	6.7± 2.2
*Cancer type*	
Lung cancer, n (%)	287 (97.6)
Malignant thymoma, n (%)	5 (1.7)
Esophageal cancer, n (%)	2 (0.7)
*Disease stage*	
IIIA	64 (21.8)
IIIB	118 (40.1)
IV	112 (38.1)
*Tumor Pathology*	
Squamous cell carcinoma, n (%)	290 (98.6)
Malignant thymic carcinoma, n (%)	4 (1.4)
*Previous treatments:*	
Surgery, n (%)	9 (3.1)
Systematic Radiotherapy, n (%)	8 (2.7)
Chemotherapy, n (%)	205 (69.7)
Immunotherapy, n (%)	2 (0.7)
Lymphocyte (%,mean ± SD)	21.7± 8.6
*ECOG Performance Score (PS)*	
2	11 (3.7)
3	194 (66.0)
4	89 (30.3)

SD, standard deviation.

### Local Single Needle Cone Puncture-^125^I seed (SNCP-^125^I) Brachytherapy Procedure

To achieve an accurate and dosimetric distribution of ^125^I-seed implantation, treatment-planning system was applied for the ^125^I treatment (TPS; standard version; Beijing ASTRO Technology Development Co., Ltd.) based on the American Association of Physicists in Medicine TG43 brachytherapy formalism (21–25). Dose, seed distribution, and depth of needles of each patient were determined preoperatively *via* TPS (**Table 2** and **Supplemental Figure 2**). Dosimetric evaluation parameters were: 1) D90, dose covering 90% tumor volume; 2) V90, tumor volume covered by 90% dose; 3) V100, tumor volume covered by 100% dose; and 4) V150, tumor volume covered by 150% dose. V90 ≥90% was considered as adequate dosage and distribution. Under general anesthesia, patients lay down in appropriate operational positions and an intraoperative stereotactic CT was performed. Thereafter, puncture paths were selected according to the preoperative planning under the CT scanning with a slice thickness of 5mm and seed brachytherapy of each needle channel was completed using 18G needle (15–20 cm\18G- needle, Zhuhai Hejia Inc., China). Enhanced CT scan was generally applied during operation due to the complicated structure of mediastinum and lung hilar. Seeds distribution and dose was verified through TPS after operation, and there is no significant difference between TPS predicted and implanted dose (**Table 2**). ^125^I seeds used in this study was 4.50 ± 0.3 mm long, sealed and covered with an envelope of nickel titanium alloy (Atomic High-Tech Co., Ltd., Beijing). It is with an outer diameter of 0.80 ± 0.03mm, half-life of 59.6 days and an activity of 0.8 mCi. Summary of implanted ^125^I seed parameters was shown in **Table 3**.

**Table 2:** Comparisons of seed parameters between TPS prediction and implanted dose.

Parameter	TPS prediction (n=294)	Implanted (n=294)	*p* value
D90 (cGy)	10490 ± 87.98	10570 ± 88.34	0.496
V90 (%)	95.67 ± 0.13	95.64 ± 0.13	0.906
V100 (%)	91.25 ± 0.10	91.19 ± 0.10	0.635
V150 (%)	75.42 ± 0.27	75.31 ± 0.26	0.777

TPS, Treatment Planning System; D90, Dose covering 90% tumor volume; V90, tumor volume covered by 90% dose; V100, tumor volume covered by 100% dose; V150, tumor volume covered by 150% dose. Data shown as mean ± SEM. Student t test was used for comparisons between groups.

**Table 3:** Overall I^125^ seeds implantation parameters.

Parameter	Mean ± SD (n=294)	Range
Total radiotherapy dose (cGy)	11027.2 ± 1502.9	8000-14000
Individual seed activity (mCi)	0.8	0.8
Total seeds activity (mCi)	44.4± 16.6	10.4-151.2
Number of seeds implanted	55.2± 20.9	13-189
Total puncture channels	5.0 ± 1.6	3-10
Number of cones formed	3.0 ± 1.6	1-8
Implanted seeds parameters		
D90 (cGy)	10573.0 ± 1514.7	7978.7-13084.4
V90 (%)	95.6 ± 2.2	85.3-98.0
V100 (%)	91.2 ± 1.6	82.1-93.0
V150 (%)	75.3 ± 4.5	67.2-83.0

D90, dose covering 90% tumor volume; V90, tumor volume covered by 90% dose; V100, tumor volume covered by 100% dose; V150, tumor volume covered by 150% dose.

Particularly, we developed the Single Needle Cone Puncture method for the ^125^I seed implantation (SNCP-^125^I). We first selected one puncture site on the skin, withdrew the needle upon completion of ^125^I seed implantation in the first needle channel until the needle tip was 1 to 2 cm from the tumor, then adjusted puncture direction avoiding blood vessels and performed the second puncture with the needle still inside the thoracic cavity. Similarly, the third or fourth puncture path was conducted inside the thoracic cavity according to the tumor shape, size and TPS plan, the needle direction was adjusted inside the thoracic cavity. Three needle channels form a three-dimensional cone shape covering over 90% of tumor volume. The end of each needle channel was close to be parallel with a minimum distance of 1.5cm between each two adjacent needle tracks to ensure the distribution of radioactive seeds covering most of the tumor area. Further, tumor with a diameter of 3 to 4 cm usually can be covered with one three-needle channel formed cone structure. It can be completed with several separate cones or superimposed cones when tumors are larger than 4cm according to the shape of tumors. The ultimate goal is to make sure the radioactive sources as evenly distributed in the tumor as possible under the premise of patient safety. It is worth to note that, we always try to keep a minimal distance of 1.5 to 1.7 cm of the seeds and normal tissues/organs to limit radiation dose outside the tumor and avoid radioactive complications.

### Tumor Measurement and Observation of Therapeutic-Related Side Affects

CT scans were performed to measure tumor size pre- and 3 to 6 months post treatment. Tumor response evaluations were conducted according to the Response Evaluation Criteria in Solid Tumors (RECIST version 1.1) guidelines. Tumor size was measured by the sum of biggest diameter of all target lesions. Objective responses were defined as follows: CR, complete response; PR, partial response, described as a 30% decrease in the biggest diameters of all targeted tumors; PD, progressive disease, defined as new tumor appearance or a minimum 20% increase in the biggest diameters of all targeted tumors; and SD, stable disease, determined as tumor change between PR and PD. Procedure related side effects were recorded during treatment and follow-up according to Common Terminology Criteria for Adverse Events (CTCAE) version 4.0 (26). Disease-related symptoms before treatment were not disclosed unless they worsened after ^125^I brachytherapy.

### Hematoxylin and Eosin (H&E) Staining and Immunohistochemical Analysis

Tumor core biopsy tissues pre- and post- treatment were fixed with 10% neutral formalin, dehydrated, embedded and serially sectioned (4 µm thick) for H&E staining using an automatic linear slide stainer (BOND-MAX) to assess pathological changes after treatment. Pathological diagnosis was made by two independent blinded pathologists. All sections were immunohistochemically (IHC) stained with p40 (Cat. No.:ZM-0472, clone# BC28) and P63 (Cat. No.: ZM-0406, clone# 4A4 + UMAB4) *via* automatic IHC stainer (BOND-MAX). Second antibodies are provided by Leica Biosystems Co., Ltd. p40, p63, CD8+, and Ki67-positive cells were stained for brown nucleus. To count CD8+ and Ki67 positive cells, the richest positive cell areas were identified at low magnification (×10) were selected, images were took at a magnification of ×20, and then CD8+ and Ki67 positive cells were evaluated quantitatively by two independent observers who analyzed five fields from these areas under a high-power (×40) field using an Olympus confocal microscope (Center Valley, PA, USA).

### Follow-Up Assessment

All patients were followed from the date of the treatment of SNCP-^125^I brachytherapy up to September 2020 or up to the time of death. Treatment-related adverse events were recorded during the treatment and follow-up.

### Statistical Analysis

All statistical analyses were performed using the GraphPad Prism 5 (GraphPad Software Inc., La Jolla, CA). Survival curves and rates were calculated using the Log-rank (Mantel-Cox) Test or Gehan-Breslow-Wilcoxon Test, and survival was measured from the date of the treatment of local radioactive Iodine-125 seed brachytherapy up to September 2020 or the time of death. A student t test was used to analyze the statistical significance between groups. Spearman test was used to analysis correlations between groups. A *p* value less than or equal to 0.05 was the threshold used to determine statistical significance.

## Results

### Clinical Outcomes of SNCP-^125^I Brachytherapy-Treated Patients With Incurable Thoracic Malignancy

**Figure 1:**
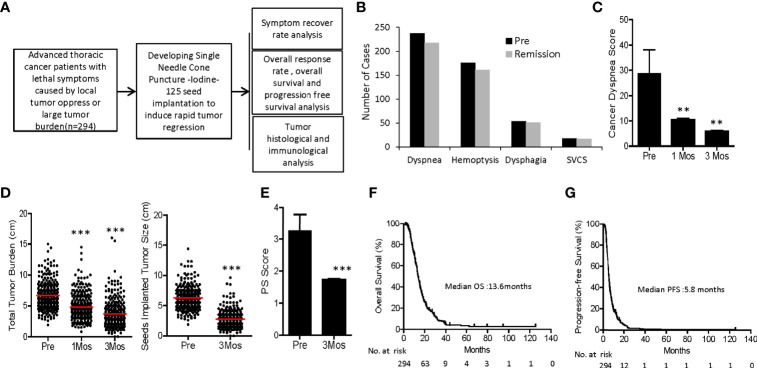
Flow chart and clinical outcomes of SNCP-^125^I brachytherapy-treated patients with incurable thoracic malignancy. **(A)** An overall workflow of the present study. **(B)** Hemoptysis and life-threatening symptoms including dysponea, dysphagia and superior vena cava syndrome due to local tumor oppressions were greatly alleviated in 2 weeks to 1 month. **(C)** Cancer dysponea score was significantly decreased 1- and 3-month after treatment. Cancer dysponea score was calculated according to the widely used Cancer Dyspnoea Scale reported by Tanaka et al. (27). **(D)** Overall tumor burden and change of tumor size after treatment in 294 patients 1 month and 3 months post SNCP-^125^I brachytherapy. 26 patients showed partial response (PR), 1 patient with complement response (CR) and 3 patients with stable disease (SD). **(E)** Performance status of all 294 patients was greatly improved following SNCP-^125^I brachytherapy. **(F, G)**. Overall survival (OS) and progression-free survival (PFS) curves of 294 patients. The median OS and PFS were 13.6 months and 5.8 months, respectively. Survival curves were analyzed using the Log-rank (Mantel-Cox) Test. SVCS, superior vena cava syndrome. *p < 0.05; **p < 0.01; ***p < 0.001.

We investigated 294 patients with advanced thoracic malignancy who were diagnosed as squamous cell carcinoma and progressed on multiple types of conventional treatments (**Figure 1A**). Particularly, 238 of the patients experienced dyspnea, 54 dysphagia, 18 super vena cava syndrome due to local tumor oppressions and 176 hemoptysis. A high proportion of patients developed two or more types of the above symptoms (**Supplemental Figures 3**, **4**). Following SNCP-^125^I treatment, the symptoms were successfully alleviated in 91 to 94% patients (**Figures 1B, C**). Of note, all the patients showed limited anatomical puncture site that restricted to traditional multiple parallel needle-^125^I seed brachytherapy. Following the SNCP-^125^I treatment, which we implement a radioactive dose covering over 90% of the tumor volume using a single needle through one puncture site on the skin, the overall response rate (ORR, including PR and CR) in 3 months was 81.0% (238/294) with significantly regressed tumors and improved performance status (**Figures 1D, E** and **Table 4**). Patients with large irregular and necrotic tumors also show clinical improvements (**Figure 1D**). Of the 294 patients, 117 patients underwent chemotherapy, 2 patients followed with immunotherapy and 175 patients opted to receive hospice cares 3 to 6 months post SNCP-^125^I treatment (**Table 4**). The 1-, 2-, 3- and 5- year overall survivals of these patients were 60.2%, 18.5%, 7.7% and 2.7%, respectively. The median OS and PFS were 13.6 months and 5.8 months (**Figures 1F, G**). Procedure-related side effects of pneumothorax (32/294), blood-stained sputum (8/294), subcutaneous emphysema (10/294), puncture site bleeding (16/294) and chest pain (6/294) were observed (**Table 4**). These results suggest that, as a further local treatment opportunity, SNCP-^125^I brachytherapy can effectively and safely treat incurable thoracic malignancy, particularly for patients with inaccessible puncture sites, complications from tumor oppression and tumor bleeding.

**Table 4:** Clinical outcomes and therapeutic-related side effects of SNCP-^125^I brachytherapy treated patients.

Parameter	Patients (n=294)
Clinical response at 2-6Mos follow-up	
Complete response, n (%)	2(0.7)
Partial response, n (%)	236 (80.3)
Stable disease, n (%)	45 (15.3)
Progressive disease, n (%)	11(3.7)
Procedure-related side affects	
Pneumothorax, n (%)	32(10.9)
Blood-stained sputum, n (%)	8 (2.7)
Subcutaneous emphysema, n (%)	10 (3.4) 1 (3.3)
Puncture site bleeding, n (%)	16 (5.4)
Chest pain, n (%)	6(2.0)
Following treatment (3-6 months after I^125^ initiation)	
Chemotherapy, n (%)	117 (39.8)
Immunotherapy, n (%)	2 (0.7)
Hospice supportive only, n (%)	175(60.2)

**Figure 2:**
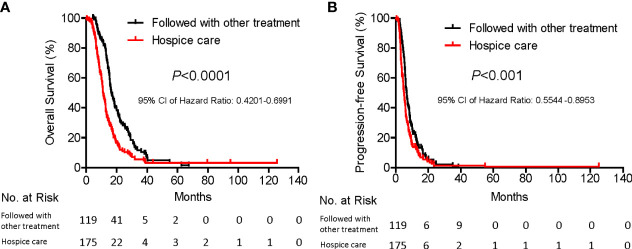
Subgroup survival analysis of patients with and without curative treatment after SNCP-^125^I brachytherapy. Among 294 patients, 119 were followed with chemotherapy and immunotherapy and 175 patients with hospice care. Patients who were followed with other types of treatment showed significantly prolonged overall survival (**A**, 16.5 months Vs. 11.2 months, *p*<0.0001) and progression-free survival (**B**, 6.8 months Vs. 5.2 Months, *p*<0.001).

Importantly, further survival analysis showed that patients who were able to follow with chemotherapy or immunotherapy experienced extended overall and progression free survival, as compared with patients who opted to receive hospice care (16.5 months Vs. 11.2 months*, p*<0.0001; 6.8 months Vs. 5.2 Months*, p*<0.001, respectively, **Figure 2**). Basic clinical characteristic factor comparisons of these two groups showed no significantly difference (**Table 5**). We also show greatly reduced risk associated with the operation and satisfied recovery rate upon treatment, especially in patients with lung hilar and mediastinum tumors. To further explain how the SNCP-^125^I approach provides an optimal clinical benefit for patients, we show the detailed treatment process for representative patients below by different clinical symptom categories.

**Table 5:** Clinical and demographic characteristic comparisons of two groups’ patient at baseline.

Characteristics	Patients followed with hospice care (n=175)	Patients followed with other treatment (n=119)	*P* Value
Gender (male-female)	133-42	90-29	1.000
Age (year, mean ± SD)	66.86 ± 9.56	64.33 ± 9.39	0.043
Weight (kg, mean ± SD)	64.75 ± 9.11	66.76 ± 8.92	0.051
Height (cm, mean ± SD)	168.70 ± 7.63	170.00 ± 7.29	0.149
Smoking history (yes-no)	137-38	89-30	0.485
Pleural effusion(yes-no)	21-154	14-105	1.000
Tumor size (cm, mean ± SD)	6.44 ± 1.78	6.07 ± 1.90	0.090
*Cancer type*			0.963
Lung cancer, n	171	116	
Malignant thymoma, n	3	2	
Esophageal cancer, n	1	1	
*Disease stage*			0.341
IIIA	33	31	
IIIB	73	45	
IV	69	43	
*Previous treatments:*			0.173
Surgery, n	6	3	
Systematic Radiotherapy, n	6	2	
Chemotherapy, n	146	59	
Immunotherapy, n	0	2	
Lymphocyte (%,mean ± SD)	21.03 ± 7.78	21.02 ± 7.84	0.865
*ECOG Performance Score (PS)*			0.146
2	5	6	
3	110	84	
4	60	29	

SD, standard deviation.

### SNCP-^125^I Brachytherapy Induces Rapid Tumor Regression of Large Recurring Solid Tumor After Conventional Treatment Failure

**Figure 3:**
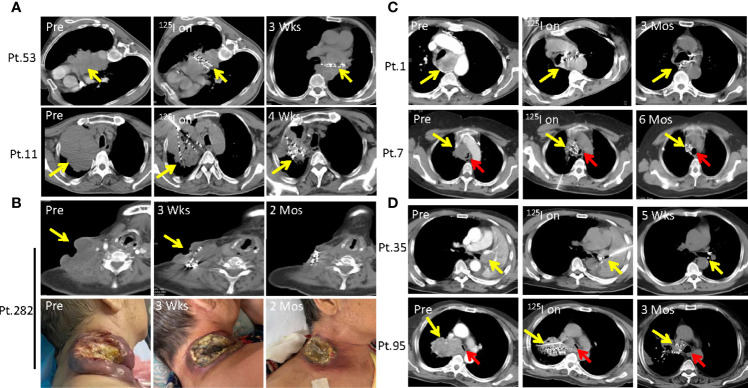
SNCP-^125^I brachytherapy induces rapid regression of large solid tumor that recurred after conventional treatment. **(A)** Patient 53 was diagnosed with large advanced mediastinal lung squamous cell carcinoma that constricted the heart and the esophagus and caused arrhythmia and dysphagia. Patient 11 progressed on chemotherapy and radiotherapy, developed a large lung squamous cell carcinoma in the left lung with no druggable targets as detected by DNA sequencing, and eventually showed a poor performance status. Tumors of Patient 11 and 53 rapidly regressed 3 and 4 weeks after SNCP-^125^I brachytherapy, respectively**. (B)** Patient 282 was diagnosed as esophageal cancer with a large metastatic and rupturing tumor in the neck. Following SNCP-^125^I brachytherapy, the tumor regressed in 3 weeks. **(C, D).** Patients 1, 7, 35 and 95 with progressive lung squamous cell carcinoma all experienced acute dyspnea because of airway obstruction. Tumors significantly regressed and the main airway was completely reconstructed in 5 weeks to 6 months. Yellow arrow-tumor, Red arrow-airway.

It is common that patients develop large lung squamous cell carcinoma and metastasis esophageal squamous cell carcinoma, and some even grow tumors on the skin surface with rupture and infection. These patients are often accompanied by serious systematic symptoms due to large tumor consumption including anemia, hypoalbuminemia, high fever and pain, with a poor performance status of Performance Score (PS) >3. Few traditional treatment strategies are currently available for these patients except hospice care. Particularly, Patient 53 was diagnosed as advanced mediastinal lung squamous cell carcinoma which compressed the heart and esophagus and caused arrhythmia and dysphagia (**Figure 3A**). Patient 11 progressed on chemotherapy and radiotherapy, developed lung squamous cell carcinoma in the left lung with no druggable targets as revealed by DNA sequencing, and exhibited a poor performance status (PS=4) (**Figure 3A**). Patient 282 was diagnosed as esophageal cancer with a large and ruptured metastatic neck tumor, and experienced severe subclavian vein oppression, edema in the right upper limb, persisting fever, anemia and low platelets levels (**Figure 3B**). We successfully administered local SNCP-^125^I treatment for these and other 84 similar patients, and found that the tumors regressed in 3 weeks (Pt.53), 4 weeks (Pt.11) and 2 months (Pt.282) respectively (**Figures 3A, B**, data not shown). The symptoms due to tumor oppression were subsequently alleviated, and the performance status greatly improved (**Figure 1E**).

### Severe Dyspnea Due to Thoracic Tumor Oppression was Completely Remedied by SNCP-^125^I Brachytherapy

**Figure 4:**
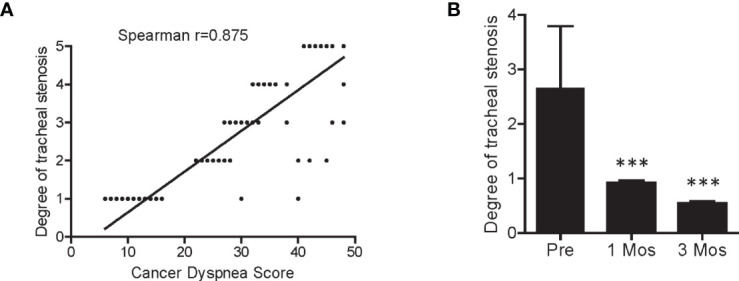
Correlation between cancer dyspnea score and degree of tracheal stenosis. **(A)** Patient cancer dyspnea score was significantly correlated with the degree of tracheal stenosis. **(B)** The degree of tracheal stenosis in patients was significantly decreased 1- and 3-month after SNCP-^125^I brachytherapy. Spearman correlation test was used for the analysis. ***p < 0.001.

Patients with squamous cell carcinoma of the lung often develop dyspnea (~60%) and acute respiratory failure, when the tumor is located adjacent to trachea, especially to the large main bronchus (28) Among the 294 patients in the present study, 238 developed dyspnea, and showed a significantly correlation with the degree of tracheal stenosis (**Figures 1B** and **4A**) (27, 29). The patients usually die in a short time if the airway cannot be recovered rapidly. However, they are unlikely to undergo conventional radiotherapy and chemotherapy as an optimal treatment strategy due to insensitivity to targeted drugs. Furthermore, the tumors are adjacent to trachea and no multiple puncture sites are readily accessible for conventional ^125^I seed brachytherapy. Consequently, rapid control and elimination of local tumor lesions represents an effective means to completely relieve the respiratory distress and severe hypoxia. As shown in **Figures 3C, D**, as representative individuals, Patients 1, 7, 35 and 95 with progressive squamous cell carcinoma of the lung all experienced dyspnea due to airway obstruction, and continued to exhibit dyspnea, orthopnea, tracheal inhalation wheeze, and declined blood oxygen saturation. Remarkably, the symptoms due to airway obstruction gradually improved in two weeks after SNCP-^125^I treatment and disappeared 1month after. The tumor significantly regressed and the main airway became significantly reconstructed in 1 to 3 months with a greatly improved cancer dyspnea score (**Figures 1C** and **4B**) (27, 29). It is worth to note that it was changeling for the patients to lie down and maintain a posture for more than 5 min due to breathing difficulties, thus limiting the operation time and requiring quick determination of the puncture site and completion of the seeds implantation within 5 to 10 min. Bleeding and severe pneumothorax during the operation will aggravate the breathing difficulty for the patients, and sufficient preparation for secondary complication and clinical emergency is needed before the operation.

### SNCP-125I Brachytherapy Successfully Alleviated Fatal Hemoptysis in Advanced Lung Cancer Patients

**Figure 5:**
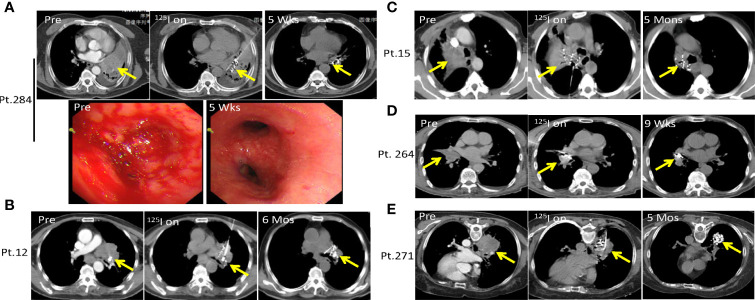
SNCP-^125^I brachytherapy successfully alleviates life-treatening hemoptysis in advanced lung cancer. **(A).** Patient 284 with squamous cell carcinoma in the hilar region of the right lung progressed on chemotherapy and developed hemoptysis, with the tumor also blocking the main trachea accompanied by dyspnea and atelectasis. Following SNCP-^125^I brachytherapy, bleeding was successfully stopped as shown under the tracheoscopy examination 5 weeks later. **(B–E)** Patients 12, 15, 264 and 271 with lung squamous cell carcinoma developed hemoptysis and progressed after unsatisfied treatment of hemostatic drugs. The tumors greatly regressed through SNCP-^125^I brachytherapy treatment in 6 months, 3months, 9 weeks and 5 months, respectively. Yellow arrow-tumor.

Hemoptysis due to lung tumors is one of the most common causes for patient death, with a mortality rate of about 59% and 80% in those with hemoptysis >1,000 ml per 24 h (30, 31). This normally happens in patients with squamous cell carcinoma developing in the central lung after ineffective standard treatment. In the present study, we utilized the SNCP-^125^I treatment to reduce local tumor burden and control bleeding in 176 lung cancer patients with hemoptysis. For example, Patient 284 with squamous cell carcinoma in the hilar region of the right lung progressed on chemotherapy and developed hemoptysis, dyspnea and atelectasis, with the tumor blocking the main trachea. Following the SNCP-^125^I treatment, the bleeding stopped as shown under tracheoscopy examination **(** **Figure 5A**). Similarly, Patients 12, 15, 264 and 271 with squamous cell carcinoma of the lung also developed hemoptysis and progressed on typical treatments and hemostatic drugs. We also effectively stopped bleeding using local SNCP-^125^I treatment which primarily reduced local tumor load and restored the trachea (**Figures 5B–E**).

### Dysphagia, Arrhythmia, and Superior Vena Cava Syndrome Caused by Mediastinal and Lung Tumors Oppressions Is Recovered by SNCP-^125^I Brachytherapy

**Figure 6:**
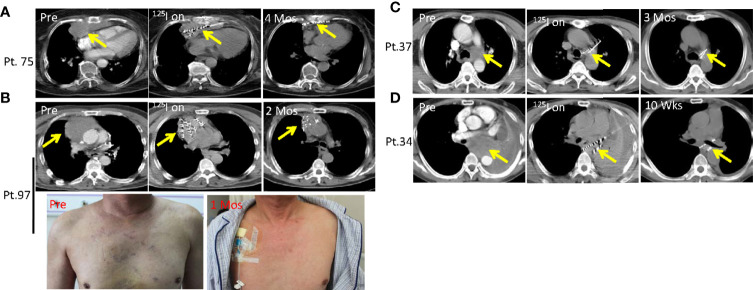
Dysphagia, arrhythmia and superior vena cava syndrome due to compression of mediastinal and lung tumors is alleviated by SNCP-^125^I brachytherapy. **(A)** Patient 75 was an 84 years old patient with malignant aggressive thymoma and progressed on surgery and radiotherapy. The tumor significantly regressed after SNCP-^125^I brachytherapy treatment in 4 months. **(B)** Patient 97 was diagnosed as malignant aggressive thymoma, experienced compression of the superior vena cava causing superficial venous dilation of the chest wall. Following SNCP-^125^I brachytherapy, the tumor significantly regressed in 2 months and superior vena cava syndrome was alleviated in 1 month. **(C)** Patient 37 with a lung tumor repressing the laryngeal nerve, invading both pulmonary artery and trachea. The tumor shrunk in 3 months *via* SNCP-^125^I brachytherapy. **(D)** Patient 34 was diagnosed as mediastinal lung cancer with the tumor compressing the esophagus and causing dysphagia. The dysphagia symptom disappeared through SNCP-^125^I treatment within a month followed by tumor regression. Yellow arrow-tumor.

Malignant aggressive thymoma is a rare epithelial tumor that often occurs in the anterior superior mediastinum. Thymoma is categorized into four stages according to the Masaoka staging system: stage I, grossly and microscopically encapsulated; stage II, the thymoma invades beyond the capsule and into the nearby fatty tissue or to the pleura; stage III, macroscopic invasion of neighboring organs; stage IV, pleural, pericardial, hematogenous, or lymphatic dissemination (32–34). Stage I and II patients generally undergo surgery, while stage III and IV patients, also called malignant aggressive thymoma, usually fail after surgical resection and require combined radiotherapy and chemotherapy. However, in some cases, aggressive thymoma quickly compresses the heart, esophagus and superior vena cava, which causes life-threatening symptoms including dysphagia, arrhythmia and super vena cava syndrome. Local SNCP-^125^I treatment is invasive and can effectively treat such tumors. Patient 75 was 84 years old with malignant aggressive thymoma progressing on surgery and radiotherapy and refused chemotherapy. The tumor oppressed the heart and caused arrhythmia which was recovered after SNCP-^125^I treatment (**Figure 6A**). Patient 97 was also diagnosed as malignant aggressive thymoma who was insensitive to radiotherapy and chemotherapy, experienced compression of the superior vena cava causing swollen head, face, and upper limbs and superficial venous dilation of the chest wall (**Figure 6B**). Following SNCP-^125^I brachytherapy, the tumor significantly regressed and superior vena cava syndrome was completely alleviated (**Figure 6B**).

Dysphagia as a result of tumor compression is another difficult-to-cure symptom. The lung tumor in Patient 37 compressed the laryngeal nerve and caused dysphagia, and experienced choking after eating. The tumor in this patient also invaded both pulmonary artery and trachea causing dyspnea (**Figure 6C**). Patient 34 was diagnosed as mediastinal lung cancer with the tumor compressing the esophagus and causing dysphagia (**Figure 6D**). Dysphagia was completely remedied in these two patients within a month after SNCP-^125^I treatment, and this was followed by tumor regression (**Figures 6C, D**). Herein, we successfully treated 54 patients who developed severe dysphagia. Taken together, the findings demonstrated that, instead of supportive care, SNCP-^125^I brachytherapy is an effective approach to reduce tumor burden when treating the life-threatening symptoms due to thoracic tumor compression.

### SNCP-^125^I Brachytherapy Is an Effective Approach in Treatment of Thoracic Malignancies With Limited Anatomic Puncture Site

**Figure 7:**
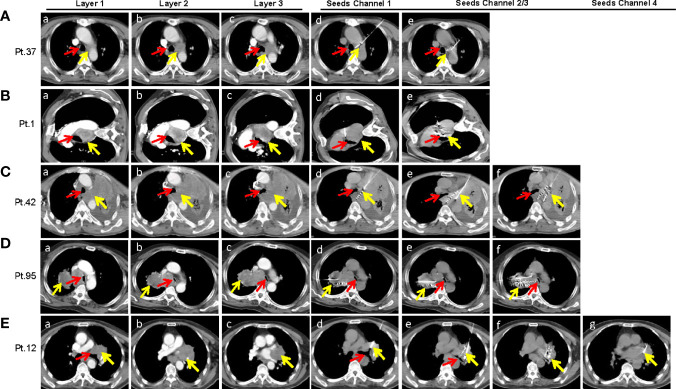
SNCP-^125^I brachytherapy is a unique approach in treating thoracic malignancies with limited numbers of accessible anatomic puncture site as shown in several representative cases. **(A)** A lung tumor in Patient 37 was surrounded by the main trachea, aortic arch and left pulmonary artery (a–c). To avoid damage to large blood vessels, only one CT layer could be the gap for needle insertion, which was less than 1 cm (c). The puncture had to pass through the whole left lung (10 cm from the tumor) that only allowed a single needle insertion (c). The first puncture was done under the aortic arch (d), and the second and third puncture through the right pulmonary artery by adjusting the needle in the aortic window without exiting the vascular space (e). **(B)** A lung tumor in patient 1 in the aortic window compressed the main trachea. The patient was only able to lie on the left side due to breathing difficulty, and this greatly limited the operation time (a–c). The first puncture was done through the intercostal space and the aortic window (d), and the second and third punctures by adjusting the direction of the needle from the edge of the diaphragm (e). **(C)** An irregular left lilar tumor in Patient 42 invaded the mediastinum, oppressed the left main trachea and the esophagus (a–c). The needle was inserted into the posterior mediastinum through the left anterior chest wall and the left hilum (d). Withdrawing the needle until 1cm from the edge of the tumor and changing puncture direction for the second punctures (e, f). **(D)** A right hilar tumor in Patient 95 invaded the mediastinum, oppressed the main trachea which was narrowed by nearly 80% (a–c). Only one needle could be inserted into the side chest wall with a total puncture path of about 20 cm to reach the distal side of the tumor because the tumor in the mediastinum that compressed the trachea was blocked by the superior vena cava and aorta (c). Both the first and second punctures reached the dorsal side of the tumor which formed a conical distribution with the third puncture (d–f). Particularly, the operation was required to be completed in 5 to 10mins due to breathing difficulty. **(E)** A left hilar tumor in Patient 12 invaded the hilar, heart and trachea (a–c). Four punctures were superimposed due to the large size of the tumor. The four punctures were done through the intercostal space (d–g). Scans of each patient were enhanced continuous CT scan with a thickness at 5mm. Yellow arrow-tumor, Red arrow-Airway.

**Figure 8:**
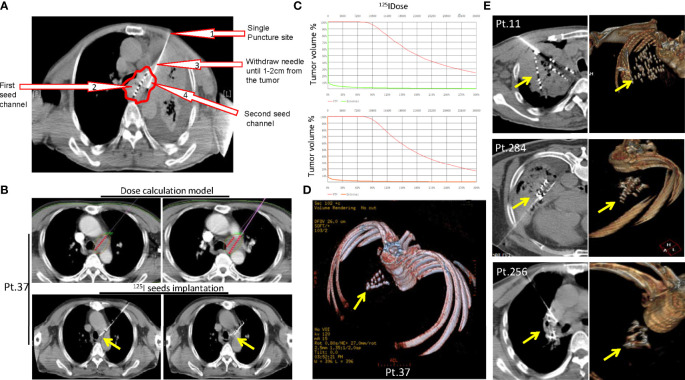
Three-dimensional cone shape was formed using a single needle.** (A)** An example CT scan showing how to operate two punctures using a single needle. SNCP-^125^I was achieved by selecting one puncture site on the skin (Arrow 1), withdrawing the needle upon completion of ^125^I seed implantation in the first needle channel (Arrow 2) until the needle tip was 1-2cm from the tumor (Arrow 3), then adjusting puncture direction and performing the second puncture (Arrow 4) with the needle still inside the thoracic cavity. Red circle-tumor. **(B)** Representative CT scans of pre-treatment radioactive dose calculation in Patient 37 through treatment planning system (TPS), and post-treatment implanted seeds paths as shown in CT scans. **(C)** Three seed paths were able to cover 90% of the tumor volume by forming a three-dimensional (3D) tapered path pre-calculated through TPS plan. **(D)** 3D reconstruction of the implanted seeds in Patient 37 showed formation of a cone shape. **(E)** Three other representative 3D reconstructed implanted seeds in Patient 11, 284 and 256 undergoing SNCP-^125^I brachytherapy. Yellow arrow-tumor.

As described above, SNCP-^125^I was effective in treating incurable thoracic malignancies. We herein highlight multiple rationales underlying the design of the SNCP-^125^I method. Foremost, tumors located in the mediastinum or hilus of the lung are normally blocked by blood vessels and trachea and thus become inaccessible to puncture. Particularly, only one accessible CT scan layer can be the puncture site with an available insertion gap of about 0.5 to 1 cm, for which typical multiple-needle puncture-^125^I brachytherapy is difficult to administer. Representative CT images of Patients 1, 12, 37, 42 and 95 were showing in **Figures 7A–E**. Secondly, patients with poor performance status require the operation to be promptly carried out, but conventional multiple needles puncture-^125^I brachytherapy usually takes over 1 h, longer than what the patients can withstand. In addition, patients cannot maintain a proper posture required for the operation because of dyspnea due to tumor oppression. Given these practical challenges, SNCP-^125^I becomes a feasible and effective approach which works though only one puncture site and can be completed in 5 to 10 min. Furthermore, for patients who were treated with bronchial stent but re-developed tracheal stenosis, SNCP-^125^I brachytherapy still restored the tracheal stenosis (**Supplemental Figure 5**). An example CT scan showing how two punctures were carried out using a single needle is shown in **Figure 8A**. Patient 37 is shown as an example to explain how the three-dimensional cone shape formed using a single needle. As illustrated in **Figure 8B**, three seed paths covered 90% of the tumor volume by forming a three-dimensional (3D) tapered path as verified through TPS plan (**Figure 8C**). Afterward, we selected one puncture site, implanted seeds into three channels by withdrawing, adjusted the direction of the needle and performed the other two punctures inside the tumor, and a 3D tapered path then formed as shown by the 3D reconstructed seeds (**Figure 8D**). As described in the method, It is important to make sure the distal ends of each channel as parallel as practical to ensure an evenly distribution of radioactive sources in the tumor. Three more representative cases of Patient 11, 256 and 284 treated with SNCP-^125^I were shown in **Figure 8E**. It was worth to note that the numbers of total puncture channels and formation of the cones were associated with the size of treated tumor **(** **Supplemental Figures 6A, B**). Taken together, SNCP-^125^I brachytherapy is an effective method for incurable thoracic cancer patients with mediastinum or lung hilum invasion and inaccessible anatomical puncture site.

### SNCP-^125^I Brachytherapy Causes Rapid and Long-Term Tumor Cell Necrosis and Induces Tumor Lymphocyte Infiltration

**Figure 9:**
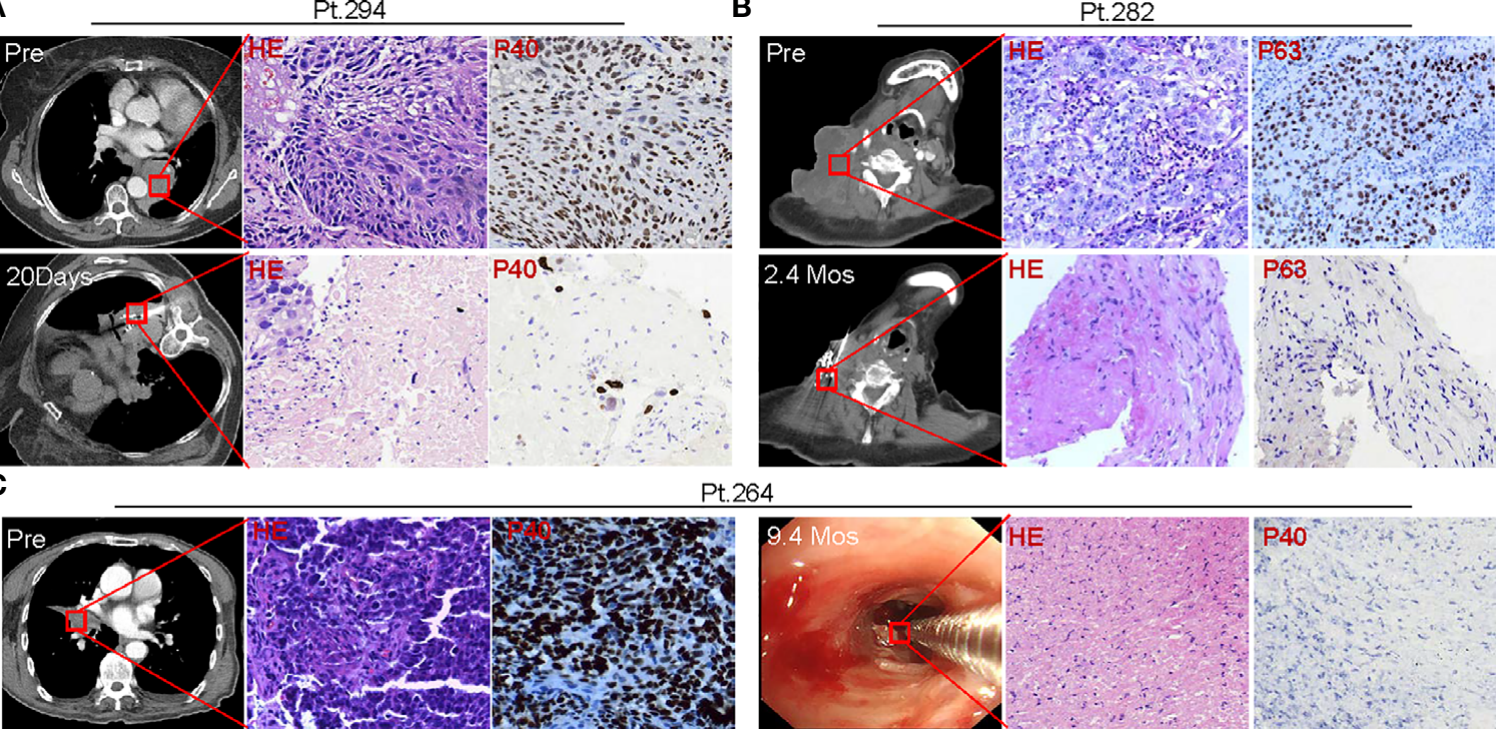
Tumor pathologic alterations following SNCP-^125^I brachytherapy. **(A)** Pre-treatment needle biopsy of the lung mass in Patient 294 showed a moderately differentiated squamous cell carcinoma, and immunostain for p40, a specific marker for squamous cell carcinoma, showed strong and diffuse nuclear expression in squamous cell carcinoma. 20 days post ^125^I seed brachytherapy, a needle biopsy within one 1cm of the seed implantation site showed scant clusters of residual cancer cells surrounded by amorphous necrotic tissue, ghost tumor cells and inflammatory cells. Residual cancer cells exhibited cytological atypia, in line with a radiation treatment effect, including enlarged hyperchromatic nuclei, multiple nuclei, cytoplasmic vacuoles. p40 staining highlights the residual cancer cells. **(B)** Pre-treatment resection biopsy of a massive metastatic cervical lymph node in Patient 282 showed poorly differentiated squamous cell carcinoma, and p63 immunostain showed strong and diffuse nuclear expression in squamous cell carcinoma cells. 2.4 months post ^125^I seed implant, a needle biopsy within one 1cm of the seed implantation site, showed spindle fibroblasts, amorphous collagen tissue, and scattered inflammatory cells. There were no visible residual cancer cells on H&E stain slides. p63 staining identified no residual cancer cells. **(C)** Pre-treatment bronchoscopic biopsy of the lung mass in Patient 264 showed poorly differentiated basaloid squamous cell carcinoma, and strong and diffuse nuclear stain pattern showed p40 expression in squamous cell carcinoma. At 9.4 months post ^125^I seed implant, a bronchoscopic biopsy at the previous biopsy site showed fibrotic tissue, great infiltration of inflammatory cells, and necrotic surface indicative of ulcer. There were no visible residual cancer cells or normal epithelial cells lining the bronchus surface on H&E stain slides. p40 staining identified no residual cancer cells. All images were taken in a 40X magnification. HE, hematoxylin and eosin staining. 8 patients were tested and 3 patients were shown here as representative cases.

**Figure 10:**
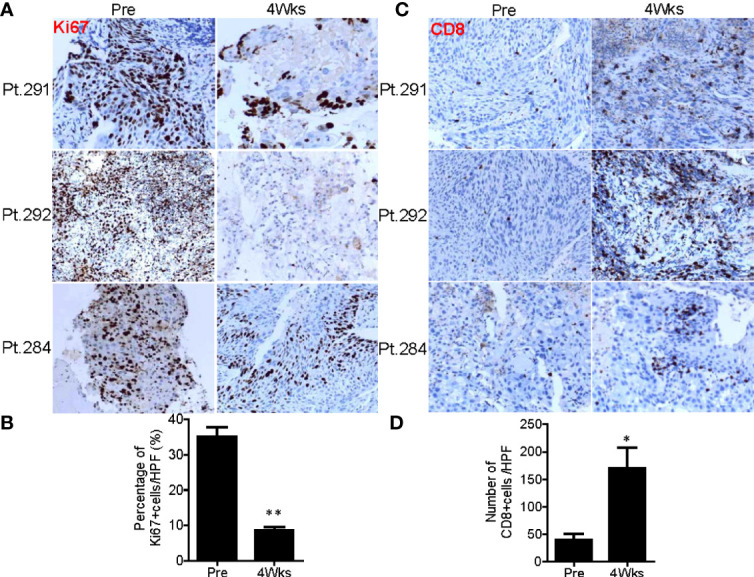
SNCP-^125^I brachytherapy enhances tumor lymphocyte infiltration. **(A)** Immunohistochemistry (IHC) staining against a tumor cell proliferation maker Ki67 in Patients 284, 291 and 292 at pre and 4 weeks post SNCP-^125^I brachytherapy. **(B)** Quantification data showed tumor proliferation was significantly inhibited after SNCP-^125^I brachytherapy (*p*=0.013, n=4). **(C)** IHC staining against tumor infiltrated CD8+T cell in Patient 284, 291 and 292 at pre and 4 weeks post SNCP-^125^I brachytherapy. **(D)** Quantification data showed a significant increase in tumor infiltrated CD8+T cells after treatment (*p*=0.043, n=4). A student t test was used to analyze the statistical significance before and after treatment. All images were taken in a ×20 magnification and zoomed out. Data were collected under a high-power (×40) field. HPF, High power field. *, p<0.05; **, p<0.01.

^125^I brachytherapy induces rapid tumor regression (**Figure 1D**) (11–17), but the pathological feature of irradiated tumor cells remains poorly defined. We thus assessed pathological alterations in tumor tissues from eight patients before and after SNCP-^125^I brachytherapy. We found that the majority of tumor cells underwent necrosis in 15 to 20 days after treatment (**Figure 9A**). After 2 or 3 months, no or little live cells were observed in the treated tumor site, which is usually difficult to confirm *via* CT scans (**Figure 9B**). Surprisingly, after 9 months, the remaining tumor cells became fibrotic (**Figure 9C**), although CT scans still show a high density shadow in the treated tumor area in most cases. All pathological alterations were further confirmed by staining with tumor specific markers p40 and p63 for squamous cell carcinoma that treated tumor fails to identify any residual cancer cells 2 to 3 months after treatment (**Figures 9A–C**). In parallel, immunohistochemistry (IHC) staining with tumor cell proliferation marker Ki67 in Patients 291,292 and 284 pre- and 1 month post- SNCP-^125^I brachytherapy showed that tumor proliferation was significantly suppressed after SNCP-^125^I brachytherapy (*p*=0.012, **Figures 10A, B** and **Supplemental Figure 7A** **)**. This result suggests that ^125^I brachytherapy induces tumor necrosis and fibrosis to eliminate tumor cells in a long-term manner, in line with the rapid tumor regression as observed through CT images (**Figure 1D**).

In vitro studies have revealed that systematic radiotherapy increases T-cell infiltration (35, 36). We therefore asked whether SNCP-^125^I brachytherapy plays a role in tumor microenvironment alteration in the patients. We found that tumor infiltrated CD8+T cells were significantly increased 4 weeks after SNCP-^125^I brachytherapy, while CD4+T cell tumor infiltration showed no change (*p*=0.043, **Figures 10C, D** and **Supplemental Figure 7B**). These results provide an additional mechanistic rationale for combining ^125^I brachytherapy with immunotherapy that modulates tumor infiltrated CD8+T cell in the clinic. However, only a small population of patients was evaluated, and increasing numbers of patients are needed to confirm this result. In addition, the dynamics of CD8+T cells infiltration remains another key question that needs to addressed.

## Discussion

^125^I brachytherapy is known to be safe and effective in advanced cancer patients (11–17), and the multiple-needle parallel puncture method is the most common and widely used treatment regimen for ^125^I brachytherapy (11–17, 37). It requires multiple accessible puncture sites without obstruction, and is suitable for tumors with relatively regular shapes. The treatment can be carried out with technical ease, and radioactive ^125^I seeds can be readily distributed evenly through multiple paralleled needles with an equal distance in between. However, critical challenges remain as it may cause severe lung injury with bleeding and pneumothorax, especially for lung hilar and mediastinal tumors (38–40). Furthermore, this approach is not applicable to larger tumors with severe irregular ulcers (**Figure 3B**), and the overall survival benefit of this treatment needs to be further determined in future controlled studies involving large populations of patients.

Herein, we present the clinical and pathological outcomes of 294 patients treated with SNCP-^125^I brachytherapy that we developed for incurable thoracic cancers. All the patients were stage III/IV thoracic malignancies, with 289 developing lung hilar and mediastinal invasion, 240 experiencing vascular and tracheal invasion, and 283 showing a performance score of 3 or 4 indicative of a high death risk. To design effective treatment strategies urgently needed to for these patients, we developed SNCP-^125^I brachytherapy which represents a highly localized treatment option and rapidly reduces local tumor burden (ORR of 81.0%), thereby significantly improving quality of life and extending treatment window for the patients (**Figure 1E** and **Table 4**). In this retrospective clinical study, we report that SNCP-^125^I brachytherapy produced a median overall survival of 13.6 months. Moreover, we found that SNCP-^125^I brachytherapy combines with chemotherapy is more efficacious than SNCP-^125^I brachytherapy alone. Importantly, we show our procedures are safe, easy to deploy, and improved quality of life for this group of patients. SNCP-^125^I brachytherapy thus represents an effective and unique treatment strategy that offers a major clinical benefit for patients with incurable mediastinal and lung hilum malignancies accompanied with life-threatening symptoms.

This SNCP-^125^I method is promising and advantageous for the following reasons: 1) multiple puncture routes can be completed with only one puncture site and one needle, and this is carried out by withdrawing and adjusting the needle direction inside the thoracic cavity; 2) it is effective treating incurable mediastinal and lung hilum tumors and large tumors with irregular ulcers; 3) it enables rapid control of life-threatening symptoms as a result of local tumor oppressions including dyspnea, hemoptysis, dysphagia and super vena cava syndrome; 4) it leads to minimal collateral tissue damage and operation-related complications (**Table 4**); 5) The procedure can be completed promptly within 5 to 10 min for patients who developed severe dyspnea; and 6) it can restore tracheal stenosis even after ineffective bronchial stent treatment (**Supplemental Figure 5**). In addition, there are several alternative approaches can be used to control the life-threatening symptoms of these patients: 1) tracheal stent placement can be used to relieve dyspnea. However, the tumor burden is not resolved, as the tumor progresses, the airway will restenosis, and the stent can also cause expectoration difficulty by continuously stimulation of the endotracheal lining. Therefore, patient’s life of quality and overall survival will not be much improved; 2) interventional vascular embolization technology can be used to control hemoptysis. Blood supply of lung tumors is usually supported by both pulmonary artery and vein. Therefore, interventional embolization is not effective as the tumor changes following treatment that will likely cause hemoptysis occurs again; 3) esophageal stent implantation is usually used to treat dysphagia. Dysphagia of these patients usually caused by mediastinal tumor compression, however, esophageal stent implantation does not treat solid tumors, but temporarily solves the eating difficult. Re-stenosis usually occurs in a short time as tumor grows, and the stent implantation will bring a lot of pain to the patients. By comparing with these approaches, the SNCP-^125^I brachytherapy treatment solves the problem of tumor compression by shrinking solid tumors in a relatively long time; thus, the curative effect, overall survival and patient quality of life are much better improved.

It is worth to note that multiple technical details must be paid attention to for SNCP-^125^I brachytherapy to succeed: 1) the puncture paths need to be design to avoid thick blood vessels and trachea, as otherwise blood vessel injury causes hemoptysis, and trachea injury causes cough and pneumothorax during and after the operation; 2) the distance between the puncture sites on the skin and the tumor should be as short as possible, which will greatly minimize the possibility of tissue injury and puncture deviation; 3) keep the needle and the predicted puncture paths relatively static following the breath floating of patients to avoid puncture deviation; 4) for new operators, can insert the needle in stages and correct in real time the direction of the needle to avoid blood vessels and trachea as found appropriate. For example, for a predicted puncture channel of 10 cm, insert the needle for 3cm to 4cm first, stop and check if the puncture direction is correct, adjust needle direction as necessary and insert another 3cm to 4cm until 10cm. It is also important to note that for lung cancer patients, the lung tissue is elastic and eligible for sufficient safe angles for needle adjustment once the tip of the needs nearly reaches to the tumor; 5) the end of each needle channel is designed to be close to be parallel with a minimum distance of 1.5cm between each two adjacent needle tracks to ensure the distribution of radioactive seeds covering most of the tumor area; and 6) the aortic window is a narrow path (normally 1 cm wide) surrounded by the aortic arch and pulmonary artery which can be used as an important path to insert into the mediastinum to avoid blood vessels.

Moreover, we assessed the impact of SNCP-^125^I brachytherapy on tumor pathological and tumor immune microenvironment, and found long-term tumor cell necrosis and fibrosis, and increase tumor CD8+T cell infiltration after the treatment. These results further provide a mechanistic rationale for further exploring combination strategies manipulating tumor-infiltrated T cells. In conclusion, SNCP-^125^I brachytherapy is a unique, feasible and effective minimally invasive therapy for patients with incurable thoracic malignancies, especially for those who develop life-threatening symptoms due to tumor oppression and exhibit limited numbers of accessible anatomical puncture sites for the conventional multiple-needle^125^I brachytherapy.

## Data Availability Statement

The raw data supporting the conclusions of this article will be made available by the authors, without undue reservation.

## Ethics Statement

The studies involving human participants were reviewed and approved by Tianjin Beichen Hospital Ethics Committee. The patients/participants provided their written informed consent to participate in this study.

## Author Contributions

FL and XD designed the study. FL wrote and prepared the manuscript with assistance and feedback of XD. FL, LW, and YZ conducted statistical plan and analyzed the data. TJ and WF collected the data. XD performed the imaging, treatments, and biopsies. ZW provided reagents or performed the histological experiments. ZL determined pathological diagnosis. XD read and measured the patient CT scans. XD provided project oversight. All authors contributed to the article and approved the submitted version.

## Funding

This work was supported by Tianjin Beichen Hospital (Grant number: Beichen District Health System Technology Project, SHGY-2020006).

## Conflict of Interest

The authors declare that the research was conducted in the absence of any commercial or financial relationships that could be construed as a potential conflict of interest.

## Supplementary Material

Supplementary Figure 1 Representative tumor pathology of patients determined by Hematoxylin and Eosin (H&E) staining. 290 of 294 patients were diagnosed as squamous cell carcinoma, and 4 as malignant thymic carcinoma. Images of other patients were not reported. All images were taken in a 40X magnification.

Supplementary Figure 2 Pre-treatment puncture design of SNCP-^125^I brachytherapy for 30 representative patients *via* treatment planning system (TPS). Puncture paths were designed following several criteria: 1) avoid thick blood vessels and trachea as practical as possible; 2) the distance between the puncture site on the skin and the tumor should be as short as possible, so as to decrease the risk of tissue injury and puncture deviation; 3) try to keep the end of each needle channel parallel with a minimum distance of 1.5cm between each two adjacent needle tracks to ensure that the distribution of radioactive seeds covers most of the tumor area; 4) the aortic window is a narrow path (normally 1cm wide) surrounded by the aortic arch and pulmonary artery that can be used as a path to insert into the mediastinum to avoid blood vessels. Magenta line-predicted puncture needle path, Red dots-^125^I seeds.

Supplementary Figure 3 Summary of life-threatening symptoms in 294 patients including the cases of one, two and three types of the symptoms.

Supplementary Figure 4 Representative CT scans of patients with two types of life-threatening symptoms. **(A).** Patient 174 with lung squamous cell carcinoma developed both dyspnea and super vena cava syndrome. CT images showed that the right trachea (yellow arrow) and super vena cava (red arrow) were severely compressed by the tumor, and this was alleviated 3 months after SNCP-^125^I brachytherapy. **(B).** Patient 211 with lung squamous cell carcinoma experienced both hemoptysis and dysphagia. CT scans showed that esophagus was compressed and the right lung hilum was invaded by the tumor, and this was alleviated in 3 months and until 12 months.

Supplementary Figure 5 An example of tracheal stenosis relapse after tracheal stent implantation in a lung cancer patient. **(A).** Relapse tracheal stenosis in Patient 250 was successfully controlled by SNCP-^125^I brachytherapy in 3 months and tracheal reconstruction lasted until 12 months (yellow arrow). Thrombosis was found in the vena cava stent (red arrow), which was not curable with collateral circulation established. **(B).** 3D reconstruction of tracheal stent (white arrow) and vena cava stent (red arrow), and tracheal stent (white arrow) was seen under bronchoscopy 12 months after treatment.

Supplementary Figure 6 The tumor size in treated patients is significantly correlated with the number of puncture channels **(A)** and the number of 3D cones formed **(B)**. Spearman correlation test was used for the analysis.

Supplementary Figure 7 Raw microscopy images on tumor core biopsy tissues staining with Ki67 **(A)** and CD8 **(B)** for **Figure 10**. All images were taken in a ×20 magnification.
