# Supplementary figures and images for: A Retrospective Study on Using a Novel Single Needle Cone Puncture Approach for the Iodine-125 Seed Brachytherapy in Treating Patients With Thoracic Malignancy
Source: Front Oncol. 2021 May 31;11:640131. doi: 10.3389/fonc.2021.640131 (PMC8200774; doi:10.3389/fonc.2021.640131)

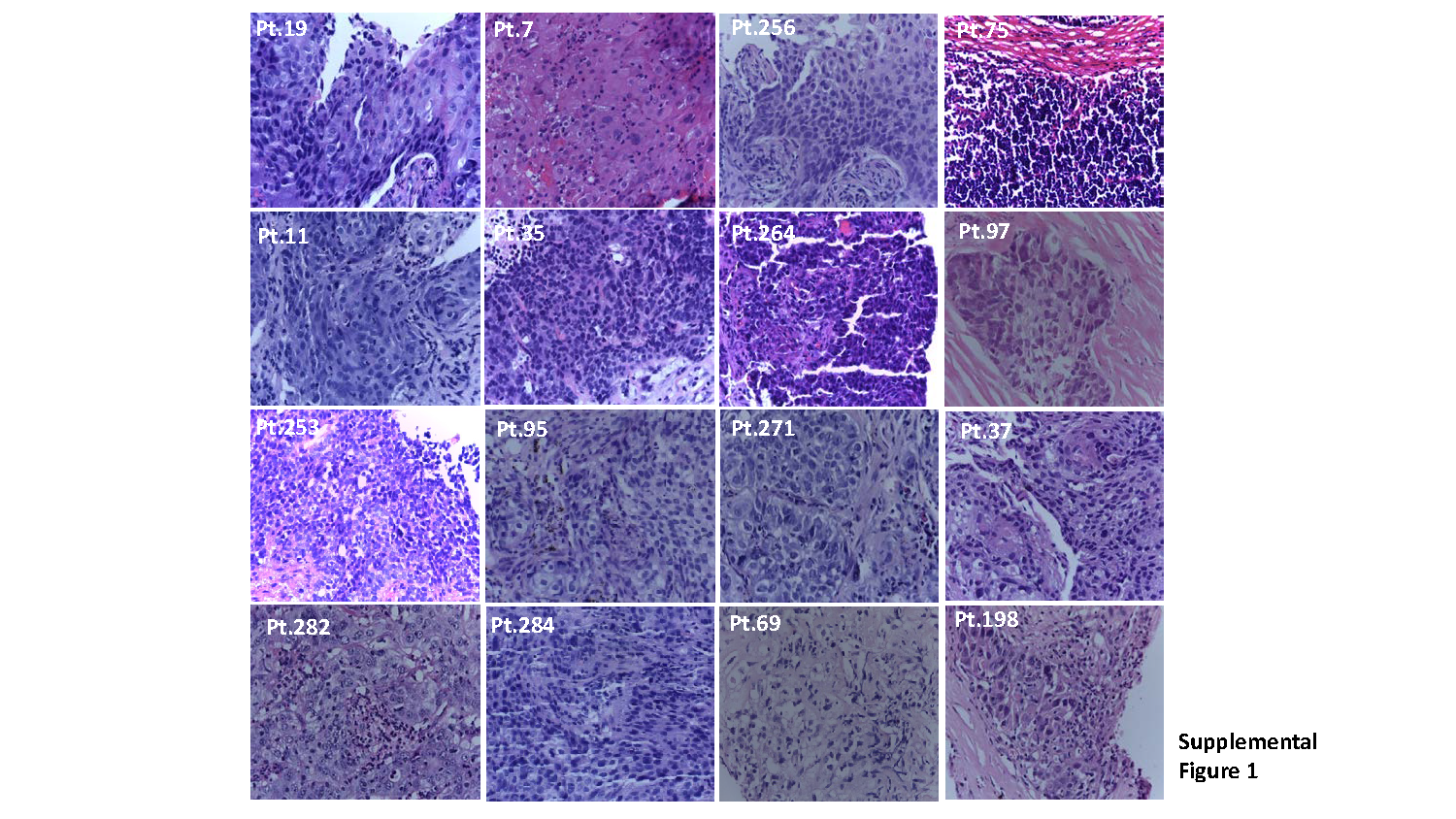

Supplement: Supplementary Figure 1 — Representative tumor pathology of patients determined by Hematoxylin and Eosin (H&E) staining. 290 of 294 patients were diagnosed as squamous cell carcinoma, and 4 as malignant thymic carcinoma. Images of other patients were not reported. All images were taken in a 40X magnification. [file Image_1.tiff]

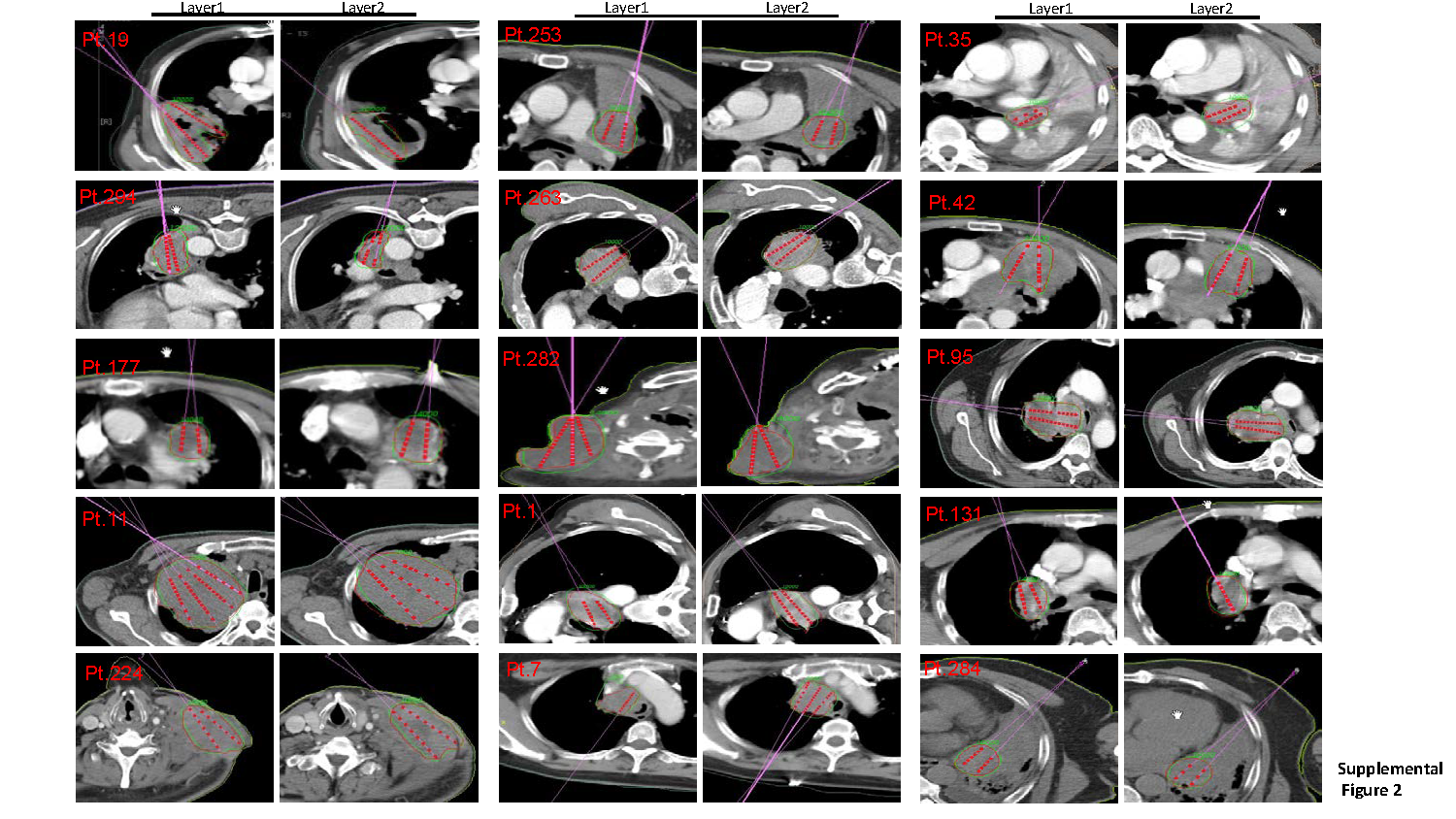

Supplement: Supplementary Figure 2 — Pre-treatment puncture design of SNCP-125I brachytherapy for 30 representative patients via treatment planning system (TPS). Puncture paths were designed following several criteria: 1) avoid thick blood vessels and trachea as practical as possible; 2) the distance between the puncture site on the skin and the tumor should be as short as possible, so as to decrease the risk of tissue injury and puncture deviation; 3) try to keep the end of each needle channel parallel with a minimum distance of 1.5cm between each two adjacent needle tracks to ensure that the distribution of radioactive seeds covers most of the tumor area; 4) the aortic window is a narrow path (normally 1cm wide) surrounded by the aortic arch and pulmonary artery that can be used as a path to insert into the mediastinum to avoid blood vessels. Magenta line-predicted puncture needle path, Red dots-125I seeds. [file Image_2.tiff]

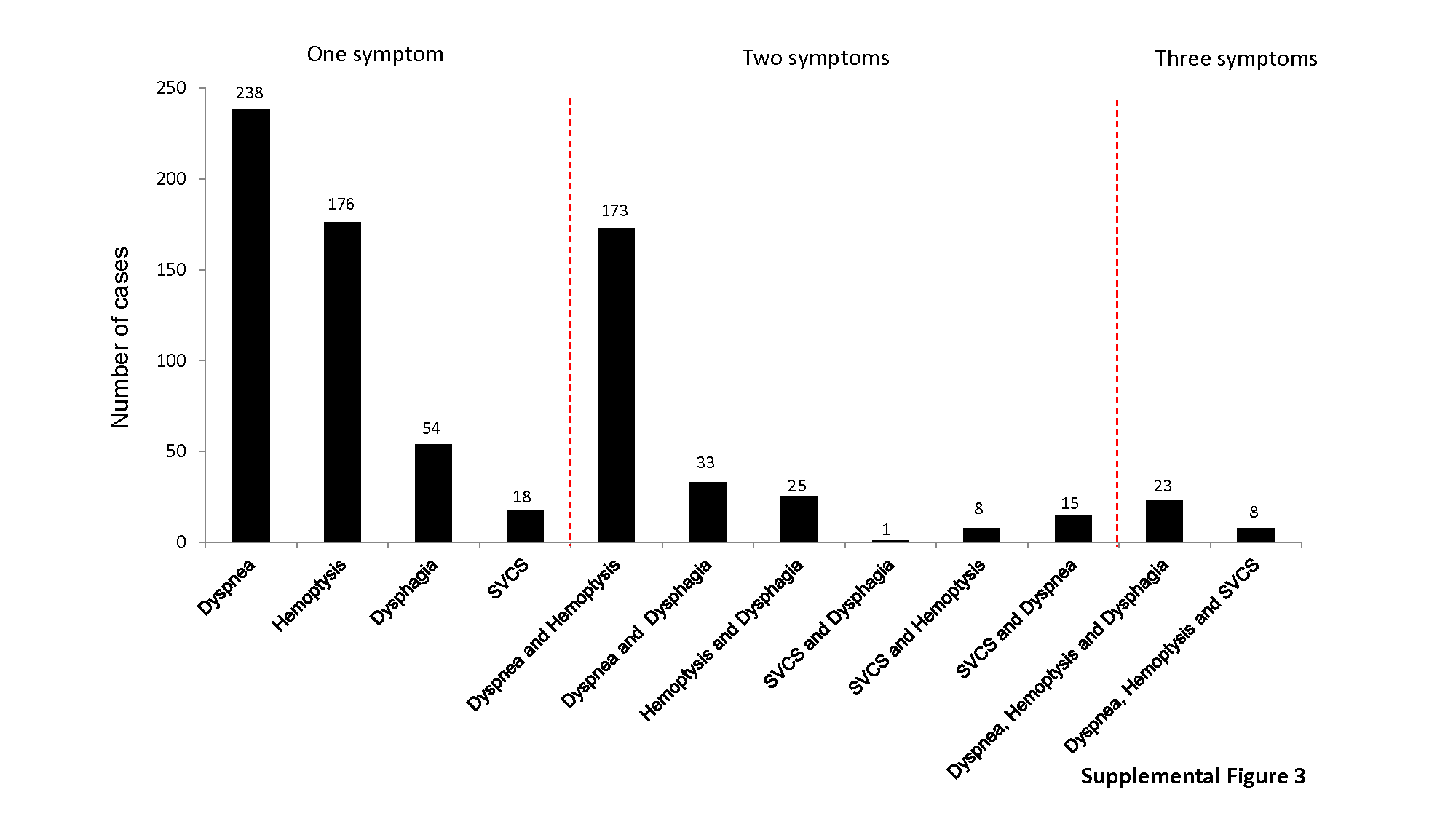

Supplement: Supplementary Figure 3 — Summary of life-threatening symptoms in 294 patients including the cases of one, two and three types of the symptoms. [file Image_4.tiff]

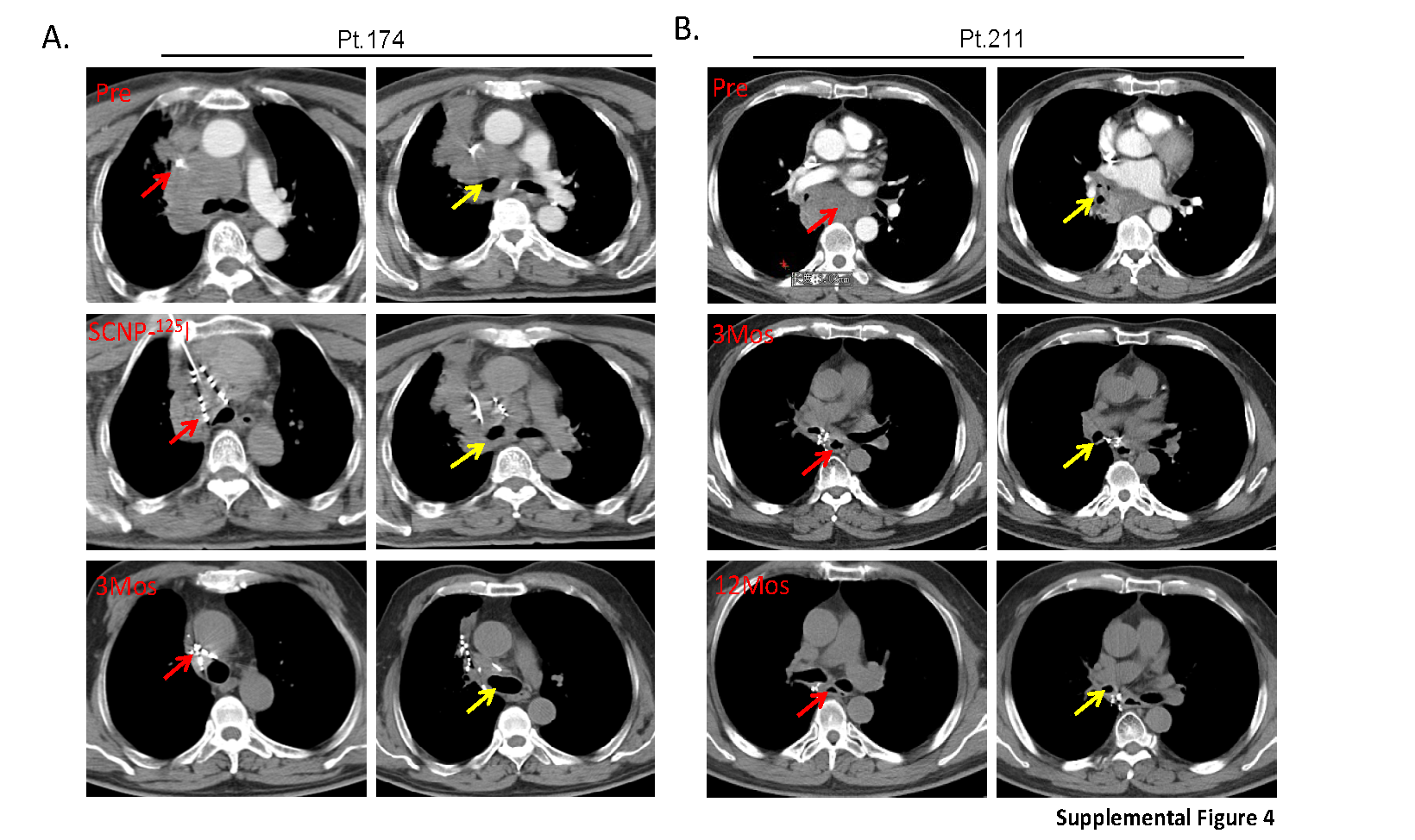

Supplement: Supplementary Figure 4 — Representative CT scans of patients with two types of life-threatening symptoms. (A). Patient 174 with lung squamous cell carcinoma developed both dyspnea and super vena cava syndrome. CT images showed that the right trachea (yellow arrow) and super vena cava (red arrow) were severely compressed by the tumor, and this was alleviated 3 months after SNCP-125I brachytherapy. (B). Patient 211 with lung squamous cell carcinoma experienced both hemoptysis and dysphagia. CT scans showed that esophagus was compressed and the right lung hilum was invaded by the tumor, and this was alleviated in 3 months and until 12 months. [file Image_5.tiff]

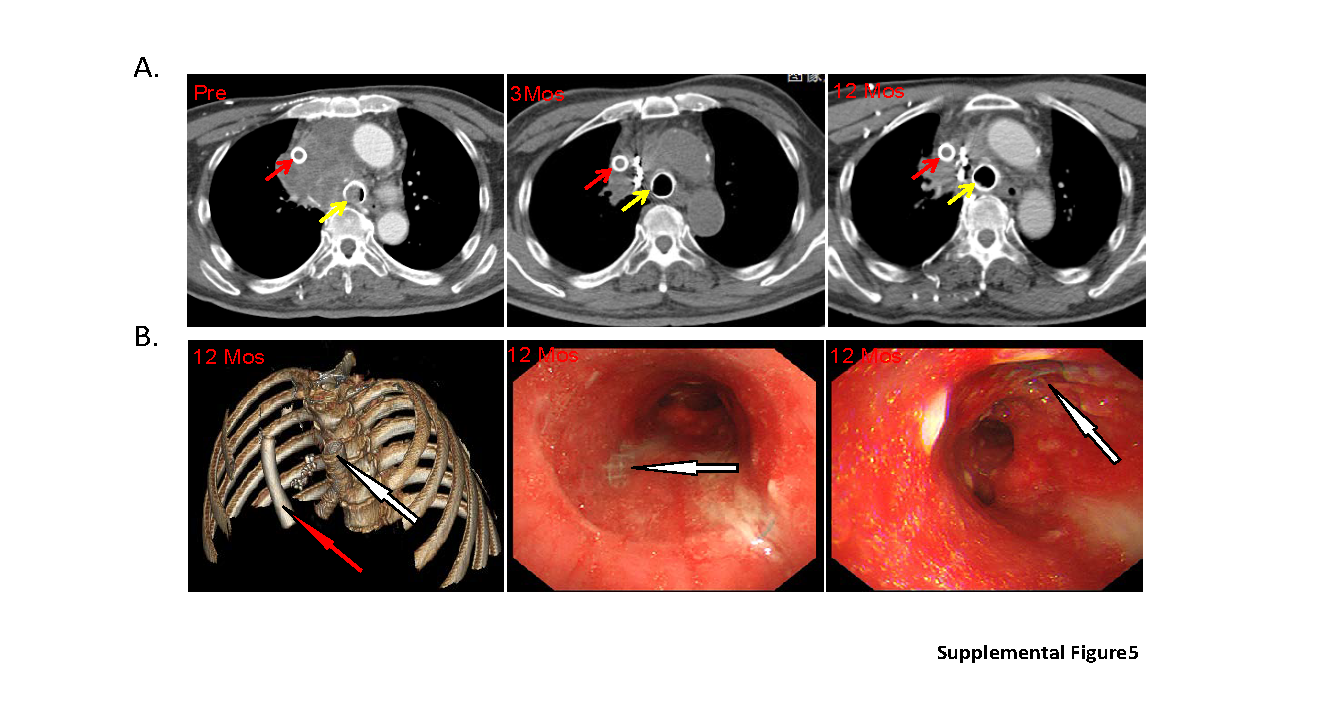

Supplement: Supplementary Figure 5 — An example of tracheal stenosis relapse after tracheal stent implantation in a lung cancer patient. (A). Relapse tracheal stenosis in Patient 250 was successfully controlled by SNCP-125I brachytherapy in 3 months and tracheal reconstruction lasted until 12 months (yellow arrow). Thrombosis was found in the vena cava stent (red arrow), which was not curable with collateral circulation established. (B). 3D reconstruction of tracheal stent (white arrow) and vena cava stent (red arrow), and tracheal stent (white arrow) was seen under bronchoscopy 12 months after treatment. [file Image_6.tiff]

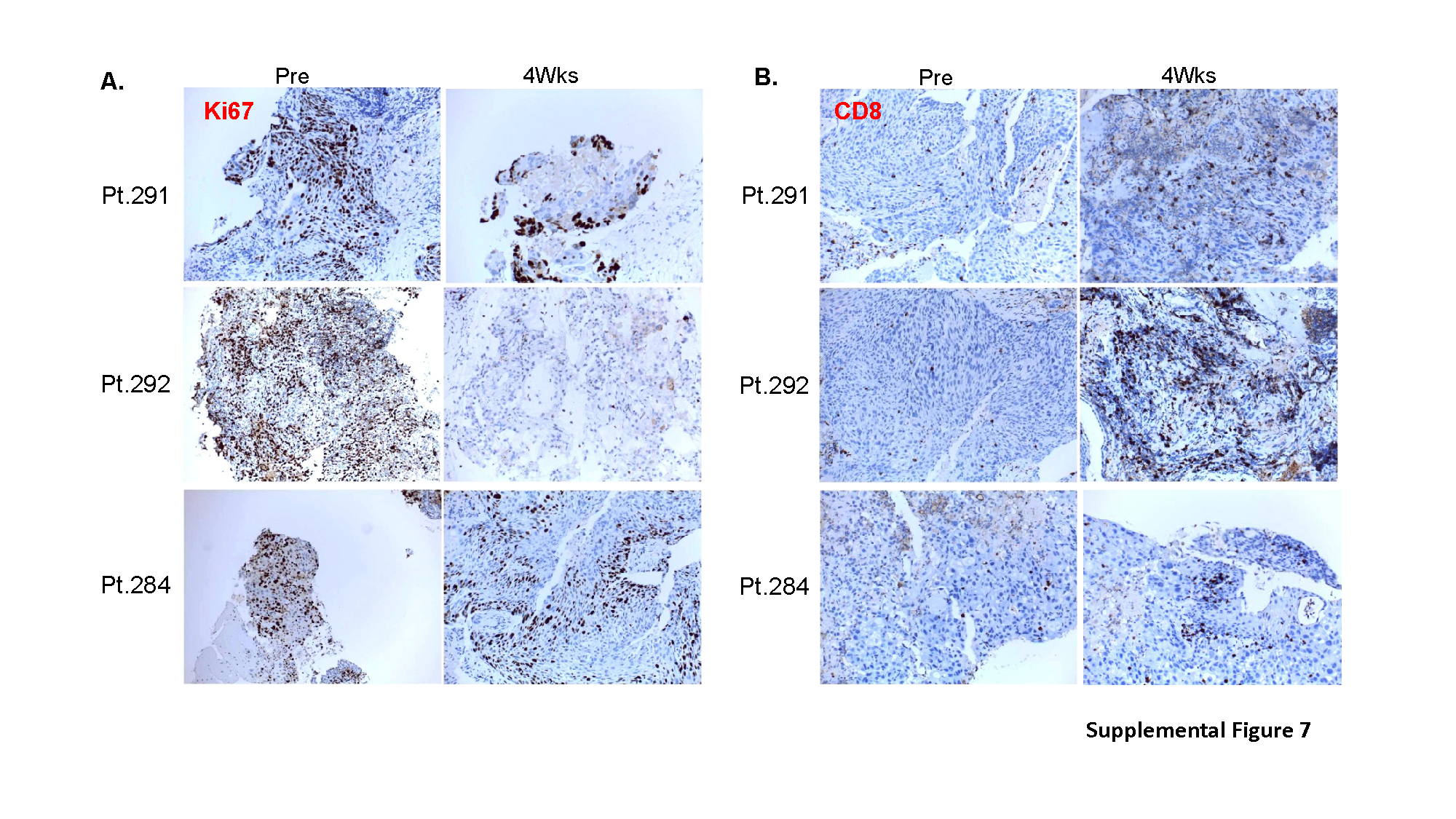

Supplement: Supplementary Figure 7 — Raw microscopy images on tumor core biopsy tissues staining with Ki67 (A) and CD8 (B) for Figure 10 . All images were taken in a ×20 magnification. [file Image_8.tiff]

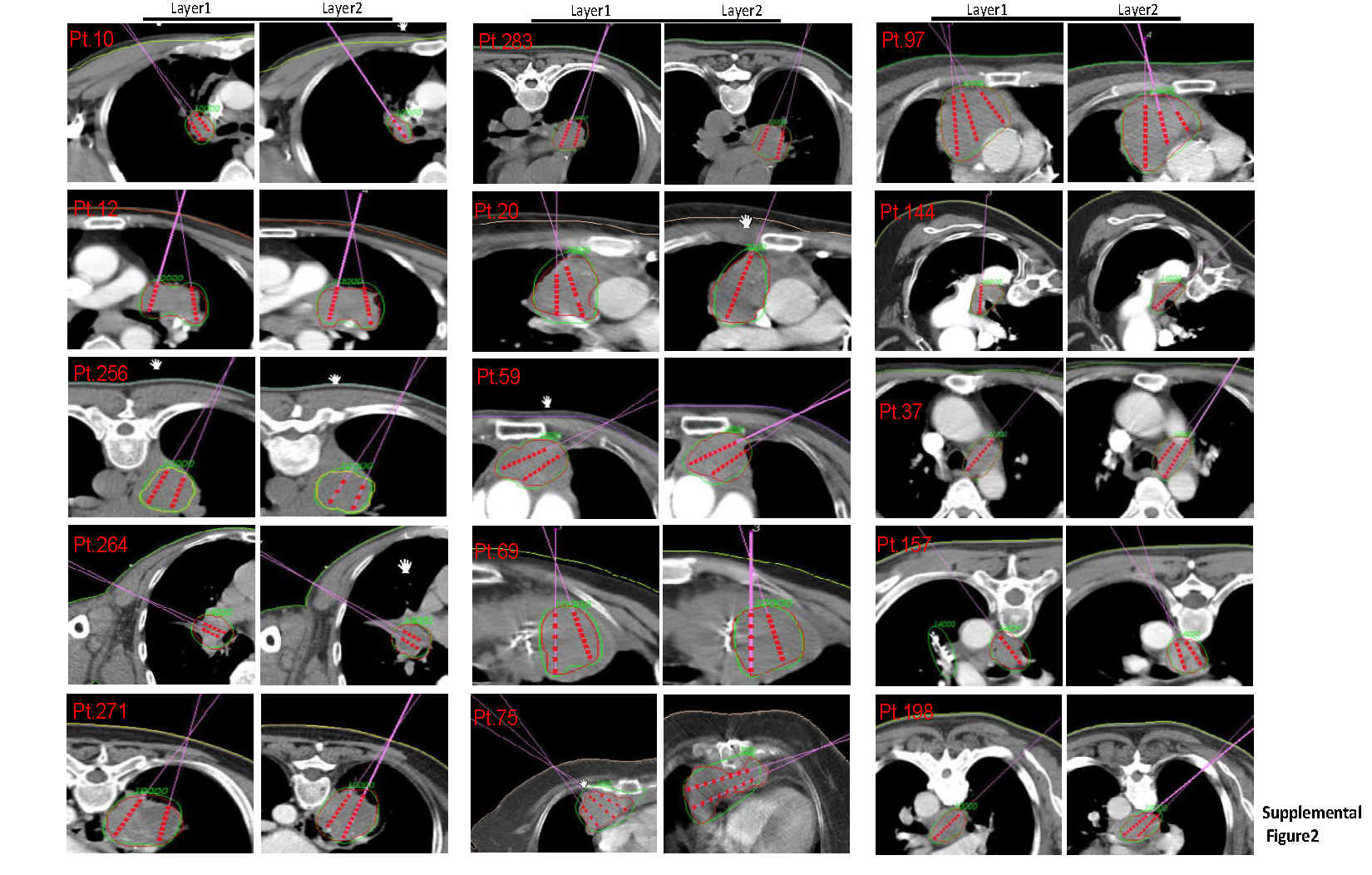

Supplement: Supplementary file 8 [file Image_3.tiff]
